# Augmented reality navigation technology in atlantoaxial pedicle screw fixation for atlantoaxial dislocation treatment

**DOI:** 10.3389/fsurg.2025.1574741

**Published:** 2025-04-25

**Authors:** Peihai Zhang, Zhenxing Sun, Kai Zhang, Jiahe Guo, Xuejun Yang

**Affiliations:** Department of Neurosurgery, Beijing Tsinghua Changgung Hospital, School of Clinical Medicine, Tsinghua Medicine, Tsinghua University, Beijing, China

**Keywords:** atlantoaxial dislocation, pedicle screw, O-arm, navigation, augmented reality

## Abstract

**Objective:**

This study aims to evaluate the clinical safety and feasibility of augmented reality (AR) navigation technology in atlantoaxial pedicle screw placement.

**Methods:**

From May 2024 to December 2024, 20 patients with atlantoaxial dislocation undergoing internal fixation were enrolled. During surgery, a real-time CT scan was obtained using an O-arm imaging system, which was processed by the navigation workstation to generate AR images. These AR images can be overlaid directly onto the surgeon's field of view, guiding him to complete pedicle screw placement. The clinical feasibility and safety were evaluated based on operative time, user experience, and the Gertzbein-Robbins scale.

**Results:**

All 20 patients successfully underwent surgery, with a total of 80 pedicle screws placement All screws met clinical safety standards, and no severe complications were observed. The operative time ranged from 16 to 21 min, with an average implantation time of 104 s per screw. The average user experience score was 90.5 points.

**Conclusion:**

This study preliminarily validates the clinical value of AR navigation technology in atlantoaxial pedicle screw fixation, supporting further investigation.

## Introduction

Atlantoaxial dislocation is a highly clinically challenging spinal surgical condition. Patients may experience numbness and weakness in their limbs, difficulty swallowing, severe respiratory failure, and even death in extreme cases. In 1994, Goel and Laheri (1) attempted to use screws and plates for the internal fixation treatment of patients with atlantoaxial dislocation. To this day, posterior atlantoaxial pedicle screw fixation has become the most widely adopted surgical technique for the treatment of atlantoaxial dislocation (2, 3). The atlantoaxial region is adjacent to important structures such as the medulla oblongata, vertebral arteries, and nerve roots. The placement of pedicle screws in this area demands precise entry points and angles, resulting in a steep learning curve for surgeons. Failure in screw placement can lead to severe surgical complications and medical disputes.

To improve the safety and precision in atlantoaxial pedicle screw fixation, and to mitigate the risks of severe complications including vertebral artery and spinal cord injuries, numerous image-guided systems have been employed. Nevertheless, the overall expenses for acquiring and maintaining computer-assisted navigation systems and surgical robots are substantial. These systems necessitate considerable operating room space, intricate operations, and contribute to an increase in both the duration and cost of surgeries. Furthermore, traditional image navigation techniques oblige doctors to divert their attention and gaze towards distant monitors, impeding the efficient coordination between hand and eye movements. This, in turn, accelerates surgeon fatigue and distraction.

In contrast to traditional image guidance, AR navigation technology has the capability to superimpose virtual images onto the surgical field, enabling surgeons to rely on this intuitive guidance for precise pedicle screw placement (4–11). This advancement is advantageous in mitigating operator fatigue and attention demands, thereby potentially shortening the duration of surgery and reducing overall medical costs (12–14). Currently, there remains a dearth of research exploring the application of AR navigation technology in pedicle screw placement, particularly for patients with atlantoaxial dislocation. This study, grounded in AR navigation technology, aims to assess the safety and feasibility of atlantoaxial pedicle screw fixation for atlantoaxial dislocation patients. It has been approved by the Ethics Committee of Beijing Tsinghua Changgung Hospital. The preliminary results are as follows.

## Methods

From May to December 2024, this study enrolled a total of 20 patients, all undergoing atlantoaxial pedicle screw placement facilitated by AR navigation technology. Of these patients, 12 were female and 8 were male. The primary symptoms reported were as follows: limb weakness in 13 cases, dysphagia in 6 cases, and a combination of limb weakness with urinary and bowel dysfunction in 1 case. Preoperatively, spinal cord function was assessed using the McCormick grading system, with 1 patient at grade I, 1 patients at grade IV, 5 patients at grade III, and 13 patients at grade II.

**Figure 1:**
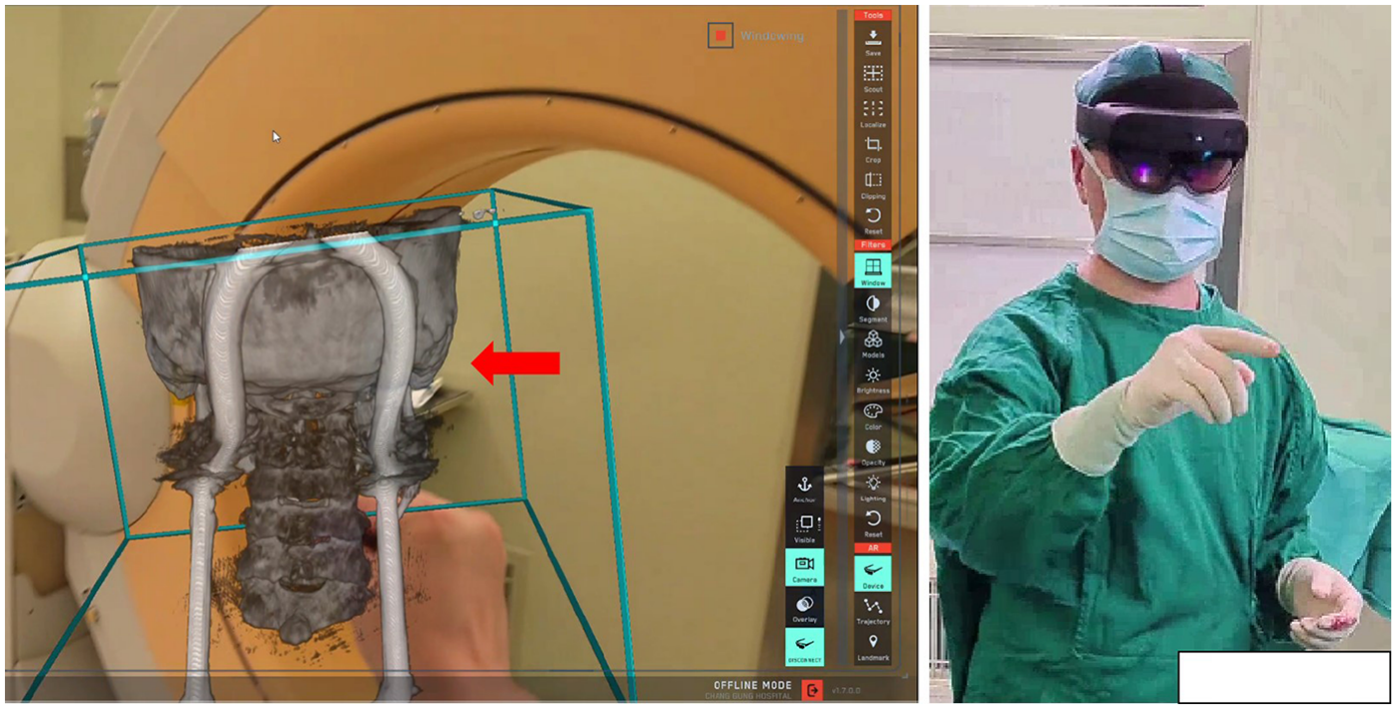
Ar image generation and browsing: the red arrow shows AR image model, which can be operated without contact after the surgeon wears AR device.

During the surgery, the patient was placed in the prone position and secured with a carbon fiber head frame. Using the traditional posterior surgical approach, the atlantoaxial lateral joint space is exposed, and a reference frame for AR image registration and tracking is fixed onto the head frame. The O-arm (Medtronic Inc., Minnesota, USA) is utilized to acquire intraoperative 3D CT images. These images are then transmitted to the AR navigation workstation (Surgical AR platform, Medivis Inc., New York, USA). After undergoing image processing, the generated AR images are sent to the AR device (HoloLens 2, Microsoft, USA). Once the surgeon wears the HoloLens 2, he can visualize the virtual three-dimensional CT reconstruction. Without needing to press any physical buttons, the surgeon can manipulate the AR images by zooming in and out, rotating, and cutting, allowing them to study and comprehend the patient's individual anatomical structure, as illustrated in Figure 1.

**Figure 2:**
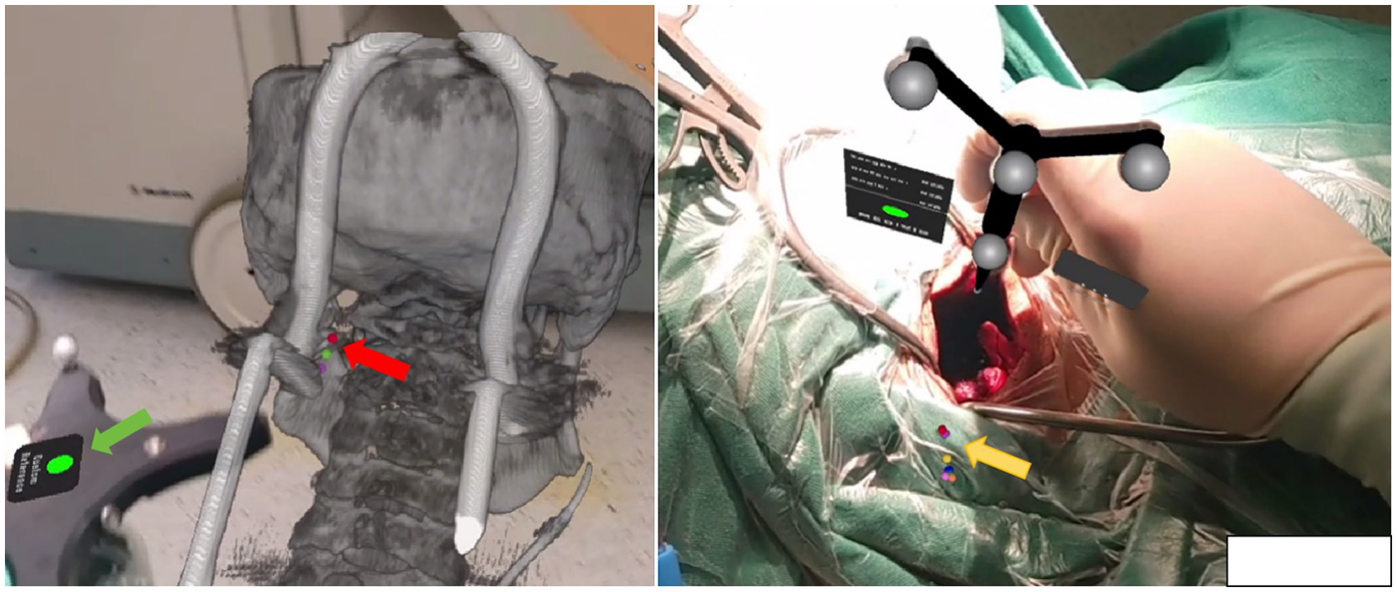
Navigation registration of AR images: red arrow shows the registration points of AR images, yellow arrow shows the registration points of real structures. The green arrow indicates the reference frame mounted on the head frame that holds the patient's head. Through the alignment of virtual and real registration points, the navigation registration of AR images and real structures can be completed.

This study employs spatial point registration technology for three-dimensional objects to achieve navigation registration between the AR virtual image and the patient's actual structure. The surgeon selects corresponding registration points on both the AR image and the real object using a navigation pointer, and the workstation is utilized to complete the spatial alignment of virtual points with real points, thereby accomplishing the navigation registration of the AR image. In this study, a surgical retractor is used as a fixed anatomical structure for point registration, as shown in Figure 2.

**Figure 3:**
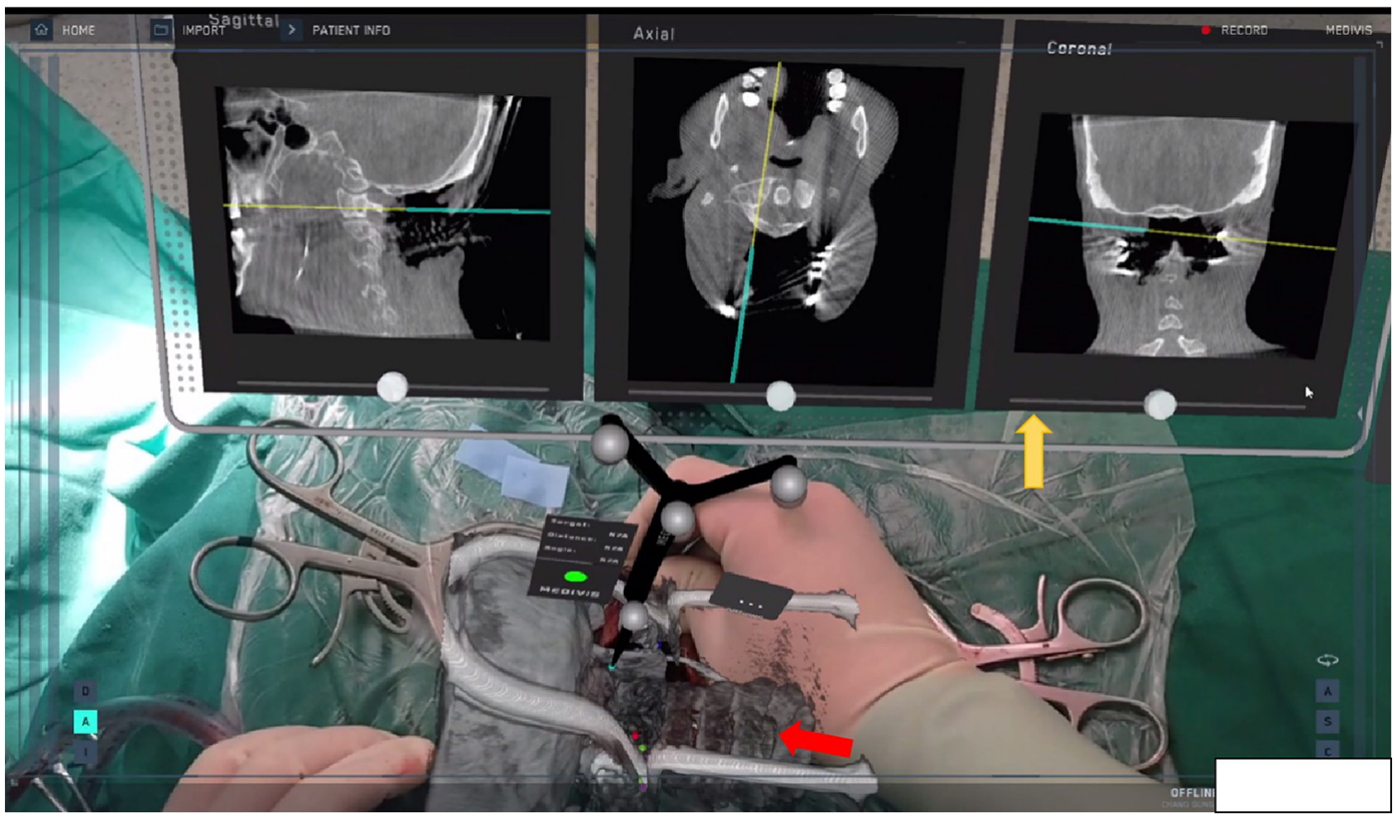
Ar navigation: the red arrow shows the *in situ* superposition of AR 3D imaging area, and the yellow arrow shows the virtual screen near the surgical area. During screw placement, the navigation pointer was used to confirm the entry points and optimal trajectories.

During the surgery, the sensors embedded within the HoloLens2 are capable of capturing the position of the infrared reflective ball on the navigation reference frame in real-time. As the surgeon's head position shifts, the spatial position of the AR image is updated in real-time. Simultaneously, throughout the surgery, specific anatomical landmarks, such as spinous processes, are intermittently employed to verify the accuracy of the navigation. Once the surgeon wears the HoloLens2, they can immediately visualize the AR 3D image superimposed onto the surgical area within their field of view. A virtual screen located near the surgical site displays information regarding the patient's sectional images. This, combined with the navigation rod, enables the surgeon to swiftly confirm the entry points and angles, as illustrated in Figure 3.

With the assistance of the AR navigation technology, surgeons completed the pedicle screw placement according to the surgical technique for atlantoaxial pedicle screw fixation. Two senior doctors used the Gertzbein-Robbins scale (15) to evaluate the safety of pedicle screw placement. If the pedicle was not broken or if it was broken less than 2 mm, it was considered safe for pedicle screw placement (grade A + B); otherwise, it was considered failure of pedicle screw placement.

Furthermore, this study conducted a questionnaire analysis to evaluate operative time and user experience, as shown in Table 1. The operative time was bifurcated into two segments: the first segment encompassed the time required for AR navigation preparation, encompassing O-arm scanning and AR image registration; the second segment pertained to the duration of pedicle screw placement. The user experience was evaluated from two main aspects, acceptability and recognition, each with a score of 50 points, totaling 100 points. The acceptability assessment investigates whether the AR device is comfortable to wear, whether the AR image can cause directional confusion, and whether the user would recommend it to peers. The recognition assessment involves whether AR devices can accelerate the surgical process and improve surgical safety.

**Table 1:** Time consumption and user experience questionnaire.

Participant	(name)	Time consumption	（s）
AR navigation user experience		Score (0–10)	
Acceptability	① Comfortable to wear		
	② AR image misdirect		
	③ Wear it all the way		
	④ Recommended peer use		
	⑤ Replace traditional technology		
Recognition	⑥ Simple and easy to use		
	⑦ Accurate positioning		
	⑧ Accelerate operation		
	⑨ Improve outcome		
	⑩ Overall satisfaction		

Descriptive statistics were used to obtain the means and standard deviations, and SPSS (version 26.0, SPSS Inc.) was used for analysis.

## Results

All the 20 patients included in this study had no obvious trauma, and the etiology was considered to be atlantoaxial dysplasia and degenerative diseases. There was no significant difference in operation time between the patients, and the surgeons were beginners in the field of spine surgery. All patients successfully underwent atlantoaxial pedicle screw placement utilizing AR navigation technology. Three leading surgeons participated in this study, and notably, no severe surgical complications were encountered. Postoperative symptoms exhibited significant improvement across all patients. Furthermore, when compared to similar cases, there was no notable increase in either hospitalization time or costs.

**Figure 4:**
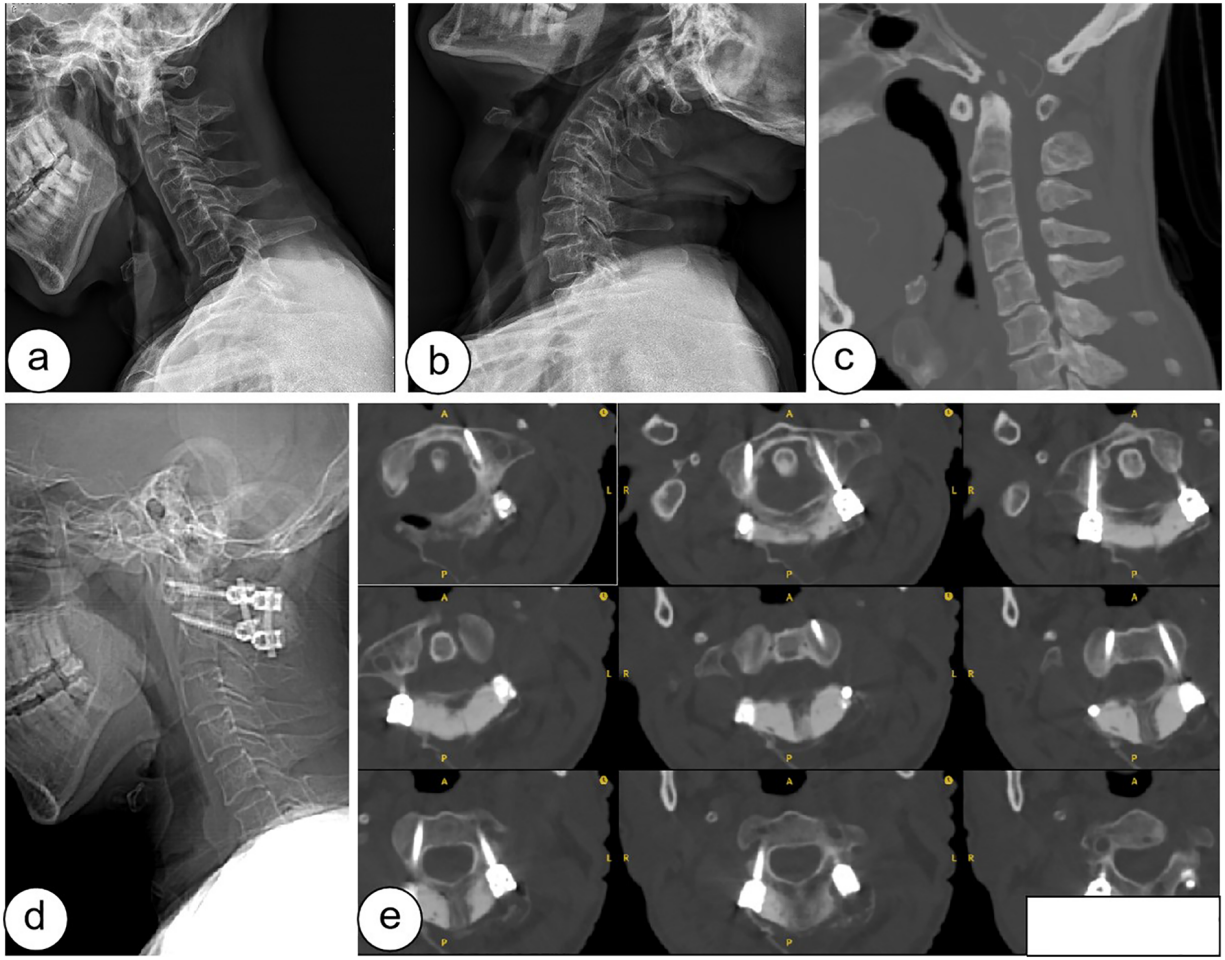
Preoperative and postoperative images of the patient: **(a-c)** are preoperative hyperextension, hyperflexion and CT of the cervical spine, **(d-e)** are lateral and CT of the cervical spine after surgery.

The surgery took between 16 and 21 min on average, with a mean of 18.5 ± 1.4 min. Among these, the O-arm scanning and AR image registration took between 10 and 1 3 min, with an average of 1 1.7 ± 1.1 min. The atlantoaxial pedicle screws placement took between 6 and 8 min, with an average of 6.9 ± 0.8 min, and each screw placement averaged 104 s. According to the Gertzbein-Robbins scale, combined with postoperative CT results, all 80 screws met clinical safety requirements, as shown in Figure 4.

Specifically, 78 screws were rated as Grade A, indicating they were completely located within the pedicle, while 2 screws were rated as Grade B, with a pedicle breach of less than 2 mm. Of these 2 Grade B screws, one case involved medial perforation and the other involved inferior perforation, as shown in Table 2.

**Table 2:** Clinical safety assessment of pedicle screw placement.

Category		Surgeon 1	Surgeon 2	Surgeon 3	Total
Number of screws		16	40	24	80
Grade A		15	40	23	78
Grade B	Above	0	0	0	0
	Inside	1	0	0	1
	Below	0	0	1	1
	Outside	0	0	0	0
Grade A screw ratio		15/16	40/40	23/24	78/80

The user experience scores given by the 3 lead surgeons ranged from 88 to 95 points, with an average score of 90.5 points. The surgeons were generally satisfied with the AR navigation technology. Specifically, the acceptance scores ranged from 43 to 46 points, averaging 45.1 points, while the recognition scores ranged from 42 to 47 points, averaging 44.8 points. These results indicate that the application of AR navigation technology in the internal fixation treatment of atlantoaxial dislocation meets the demands of clinical scenarios. The technology is simple and easy to use, capable of expediting the surgical process.

## Discussion

In comparison to conventional spinal navigation technology, AR offers a real-time, 3D image guidance during surgical procedures. This advancement markedly enhances visual intuitiveness and aids surgeons in achieving a more intuitive grasp of surgical anatomy and instrument placement. Traditional spinal navigation systems often necessitate frequent glances at remote screens by the surgeon. Conversely, AR navigation technology seamlessly integrates navigation data directly onto the surgical field of view, minimizing the need for constant diversion to distant screens. Consequently, this contributes to improved surgical efficiency and alleviates surgeon fatigue, as cited in studies (12–14).

AR technology significantly enhances the surgical experience for doctors by providing more engaging and immersive visual experiences. During surgical procedures, doctors can visualize real-time, 3D anatomical structures, select various navigation modes and perspectives, and tailor specific adjustments and settings according to individual patient anatomies to cater to a wide range of surgical requirements. The distinctive benefits of AR navigation technology have sparked a steady stream of research applications pertaining to spinal surgeries (7–10, 16–19).

Abe et al. conducted percutaneous vertebroplasty under AR navigation guidance on five patients suffering from vertebral compression fractures. Their findings revealed that the insertion angle error of the puncture needle was 2.09 ± 1.3 in the axial plane and 1.98 ± 1.8°in the sagittal plane, with notably no instances of pedicle perforation or cement leakage among the five patients (16). Additionally, Yahanda et al. executed AR-guided thoracolumbar percutaneous pedicle screw insertion on nine patients, achieving an impressive overall accuracy rate of 100% for the 63 screws placed. All screws were rated as either Gertzbein-Robbins grade A (96.8%) or grade B (3.2%) (17). In the present study, the average insertion time for each screw was 104 s, with all pedicle screws in 20 patients meeting clinical safety standards and exhibiting no screw misplacements of B-level or higher. Among the 80 screws evaluated, 97.5% were classified as Grade A, while 2.5% were classified as Grade B. The Grade B screws were related to the registration error and spinal deformation during screw placement. It is still necessary to combine surgical experience and pay attention to anatomical variation in actual operation.

Pu et al. utilized a 3D-printed navigation template for atlantoaxial pedicle screw placement in 17 patients, successfully implanting a total of 68 screws. Among these, 97.06% (66/68) were classified as Grade A screws, and 2.94% (2/68) as Grade B screws. However, the installation of the 3D-printed navigation template necessitates extensive and adequate exposure of soft tissues, leading to significant surgical trauma (20). Additionally, the template requires prior design, production, and sterilization, which increases both the cost and duration of the surgery. Zhang et al. compared the accuracy and safety of robot-assisted and navigation-assisted screw placement in atlantoaxial dislocation surgery. In the navigation group, a total of 116 screws were inserted, with an accuracy rate of 93.1% (108/116). In the robot group, 80 screws were inserted, achieving an accuracy rate of 97.5% (78/80). Nevertheless, the high cost of the equipment, the complexity of the operational process, and the requirement for specialized technical personnel make it difficult for this technology to be widely adopted in general hospitals (21).

In clinical practice, the time consumption utilizing new technology is equally crucial as user experience. This study suggest that the duration required for O-arm scanning and AR image registration varies between 10 and 13 min, averaging at 11.7 ± 1.1 min, without significantly prolonging the overall surgery time. The user experience ratings ranged from 88 to 95 points, yielding an average score of 90.5 points, which indicates an overall satisfaction with the system and its potential for further clinical application and promotion. However, it is noteworthy that 3 lead surgeons generally perceived AR images as potentially interfering with the field of view in actual surgical areas. Consequently, most operators preferred to use the device selectively, only when surgical guidance was necessary, rather than throughout the entire surgery. This suggests that there is still room for improvement in AR navigation technology research.

## Conclusion

This study demonstrates that AR navigation technology fulfills both the efficiency and precision requirements in the clinical practice of pedicle screw placement for atlantoaxial dislocations. These results are in line with clinicians' expectations for utilizing AR navigation technology to assist in pedicle screw insertion, highlighting its promising clinical application potential.

However, it is essential to acknowledge the limitations of this study, notably the relatively small sample size and the absence of evaluation for patients with severe spinal deformities. To ensure the clinical utility and feasibility of these findings, future research must validate them through larger patient populations and a more diverse range of disease types.

## Data Availability

The raw data supporting the conclusions of this article will be made available by the authors, without undue reservation.
